# Enhanced Photosynthetic Pigment Production Using a Scaled-Up Continuously Circulated Bioreactor

**DOI:** 10.3390/md21110576

**Published:** 2023-11-02

**Authors:** Won-Kyu Lee, Yong-Kyun Ryu, Taeho Kim, Areumi Park, Yeon-Ji Lee, In Yung Sunwoo, Eun-Jeong Koh, Chulhong Oh, Woon-Yong Choi, Do-Hyung Kang

**Affiliations:** 1 Jeju Bio Research Center, Korea Institute of Ocean Science and Technology (KIOST), 2670 Iljudong-ro, Gujwa-eup, Jeju-si 63349, Republic of Korea; wonkyulee@kiost.ac.kr (W.-K.L.); ykyou0111@kiost.ac.kr (Y.-K.R.); kt1024@kiost.ac.kr (T.K.); areumi1001@kiost.ac.kr (A.P.); leeyj0409@kiost.ac.kr (Y.-J.L.); iysunwoo@kiost.ac.kr (I.Y.S.); kej763@kiost.ac.kr (E.-J.K.); och0101@kiost.ac.kr (C.O.); cwy@kiost.ac.kr (W.-Y.C.); 2 Department of Marine Biotechnology, KIOST School, University of Science and Technology (UST), 217 Gajeong-ro, Yuseong-gu, Daejeon 34113, Republic of Korea

**Keywords:** *Tetraselmis* sp., photosynthetic pigment, scale-up, pilot scale, ROSEMAX

## Abstract

Microalgae have gained attention as a promising source of chlorophylls and carotenoids in various industries. However, scaling up of conventional bubble columns presents challenges related to cell sedimentation and the presence of non-photosynthetic cells due to non-circulating zones and decreased light accessibility, respectively. Therefore, this study aimed to evaluate the newly developed continuously circulated bioreactor ROSEMAX at both laboratory and pilot scales, compared to a conventional bubble column. There was no significant difference in the biomass production and photosynthetic pigment content of *Tetraselmis* sp. cultivated at the laboratory scale (*p* > 0.05). However, at the pilot scale, the biomass cultured in ROSEMAX showed significantly high biomass (1.69 ± 0.11 g/L, dry weight, DW), chlorophyll-*a* (14.60 ± 0.76 mg/g, DW), and total carotene (5.64 ± 0.81 mg/g, DW) concentrations compared to the conventional bubble column (1.17 ± 0.11 g/L, DW, 10.67 ± 0.72 mg/g, DW, 3.21 ± 0.56 mg/g, DW, respectively) (*p* ≤ 0.05). Flow cytometric analyses confirmed that the proportion of *Tetraselmis* sp. live cells in the culture medium of ROSEMAX was 32.90% higher than that in the conventional bubble column, with a photosynthetic efficiency 1.14 times higher. These results support suggestions to use ROSEMAX as a bioreactor for industrial-scale applications.

## 1. Introduction

Currently, there is a growing interest among consumers to adopt healthy eating habits and prioritize food safety [1]. In response to this trend, food manufacturing companies are making efforts to develop and industrially produce compounds from natural sources to create nutritious and safe products [2]. Furthermore, there is a global trend to discourage the use of artificial synthetic colorants [3], which has led to a growing interest in biologically synthesized pigments that are considered nontoxic [4]. Natural pigments are gaining popularity because of their potential to meet the demands of consumers seeking healthy and safe food products [5,6]. By 2028, the European natural pigment market is expected to reach USD 2.18 billion, with a compound annual growth rate (CAGR) of 10.1% [7].

Generally, natural pigments are classified into several structural types, including tetrapyrroles, carotenoids, flavonoids, curcuminoids, betalains, and others [8]. Natural pigments are derived from biological sources, including animals, plants, microalgae, bacteria, and fungi [8]. Among the organisms, microalgae are considered to be promising biomass sources for natural pigments, because of their rapid growth, high photosynthetic efficiency, high productivity of metabolites, and low greenhouse gas emissions [9,10]. The main pigments produced in microalgae are tetrapyrroles and carotenoids [8]. Tetrapyrroles are a class of pigments composed of four pyrrole units, with chlorophyll being the most common representative. In particular, chlorophyll-*a*, which is abundant in green microalgae [11], has been suggested for medical purposes including wound healing, and for antimicrobial, anticancer, anti-mutagenic, antitumor, and anti-inflammatory properties [12,13]. Carotenoids are classified as non-oxygenated carotenes and xanthophylls. The types of carotenoids include alpha-carotene, beta-carotene, lutein, zeaxanthin, and astaxanthin [14]. These carotenoids, obtained from green microalgae, have demonstrated a wide range of health benefits including disease prevention, skin protection, and anti-aging, antioxidant, and antiviral effects [15,16,17]. These findings highlight the potential of microalgae biomasses and their derived photosynthetic natural pigments as promising candidates for use in functional foods and cosmetics [18,19,20]. 

In recent years, there has been growing interest in the efficacy of chlorophylls and carotenoids extracted from *Tetraselmis* sp. As a result, research on large-scale cultivation of *Tetraselmis* sp. biomasses for the production of these pigments has been reported [21,22]. These species are primarily cultivated to produce omega-3 fatty acid-rich aquafeeds [23]. Recent research has revealed the efficacy of *Tetraselmis* sp. biomasses across a wide range of applications, including antimicrobial activity, antioxidation, metal chelation, neuroprotection, and cell recovery [19,24,25,26].

For microalgal biomass production, the most widely used cultivation system in the industry is the open raceway pond (ORP) [27]. However, open-pond cultivation often results in severe biological contamination. In the worst-case scenario, the appearance of predators can lead to culture failure within a short time [21]. For this reason, only a few species capable of growing under highly alkaline and saline-selective environmental conditions, such as *Arthrospira* and *Dunaliella*, can overcome the contamination issues [28]. Therefore, several studies have suggested that photobioreactors may be more efficient for large-scale cultivation [28] and the development of innovative photobioreactors is encouraged for large-scale microalgae production.

As the photobioreactors are scaled up in outdoor pilot-scale systems, where controlling the light can be challenging, the cell concentration tends to decrease, resulting in reduced light availability in the central region of the bioreactor. Recently, several photobioreactors have been proposed; however, most rely on artificial lighting to enhance light availability [29]. Adding artificial light can be costly and can produce heat, potentially leading to additional problems [29]. The reduction in light availability negatively affects the growth of microalgae [30]. Therefore, it is important to design a reactor with a thin structure to improve light availability. 

A bubble column bioreactor has been used as the conventional photobioreactor. Circulation is achieved using air bubbles (air injection) because stir-based mixing methods, which are generally used otherwise, are difficult to install in thin bioreactors [31,32]. The bubble column includes a rapid upward flow from the air injection points along the bioreactor axis and a downward flow near the upper wall, creating circulation throughout the entire culture medium [33]. Although this method is widely used in industry owing to its simplicity [34], relying only on bubbles can lead to the formation of partially non-circulating zones [35,36,37]. Due to these problems, closed photobioreactors encounter challenges in achieving the same level of productivity as in the laboratory during scaled-up processes [38,39]. Therefore, the development of a bioreactor that is thin—for light availability—has continuous medium circulation to prevent cell sedimentation, and uses simple air injection is necessary. The Jeju Bio Research Center of Korea Institute of Ocean Science and Technology (KIOST, Jeju Island, Republic of Korea) developed a novel, continuously circulating thin bioreactor (Korean Patent No. 1020180124656) for microalgal biomass production. This bioreactor, named Reproduction, Operation, Suspension, Easy, and Economized Max (ROSEMAX), was designed to prevent cell sedimentation by preventing non-circulating zones. Despite the fact that a previous study demonstrated that ROSEMAX could provide a stable and sufficient supply of *Arthrospira* (*Spirulina*) *maxima* biomass annually [40], more comprehensive evidence is needed to emphasize that ROSEMAX is a favorable system for microalgae cultivation. 

Therefore, this study aims to evaluate the newly developed ROSEMAX cultivation system at laboratory and pilot scales to overcome the problems of conventional bubble columns. ROSEMAX, which is thinly constructed and has a circular circulation form to reduce the light path and remove the cell sedimentation zones, will be evaluated for how advantageous it is for biomass, chlorophyll-a, and carotenoid production. To verify this, live cells in the culture medium will be selectively analyzed and photosynthetic efficiency measurements will be obtained. The results will serve as basic data for verifying the practicality of the ROSEMAX bioreactor and evaluating whether it is a viable cultivation system for scale-ups to an industrial-scale.

## 2. Results

### 2.1. Biomass Concentration and Phytochemical Composition

We analyzed the biomass concentration and photosynthetic pigment content of the cultures maintained in the conventional bubble columns and ROSEMAX bioreactors at both laboratory and pilot scales (Table 1). Statistical analyses were performed to compare the two types of bioreactors and the different cultivation scales. At the laboratory scale, no significant differences (*p* > 0.05) were observed between the two bioreactors, except for the chlorophyll-*b* content. However, at the pilot scale, both the biomass concentration and photosynthetic pigment content showed significant differences (*p* ≤ 0.05), primarily because of a substantial decrease in various factors during the scale-up of the conventional bubble column. Interestingly, with ROSEMAX, except for the chlorophyll-*b* content, no significant differences were observed in the remaining photosynthetic pigments and biomass concentrations despite the scale-up (*p* > 0.05).

### 2.2. Flow Cytometric Analysis

**Figure 1:**
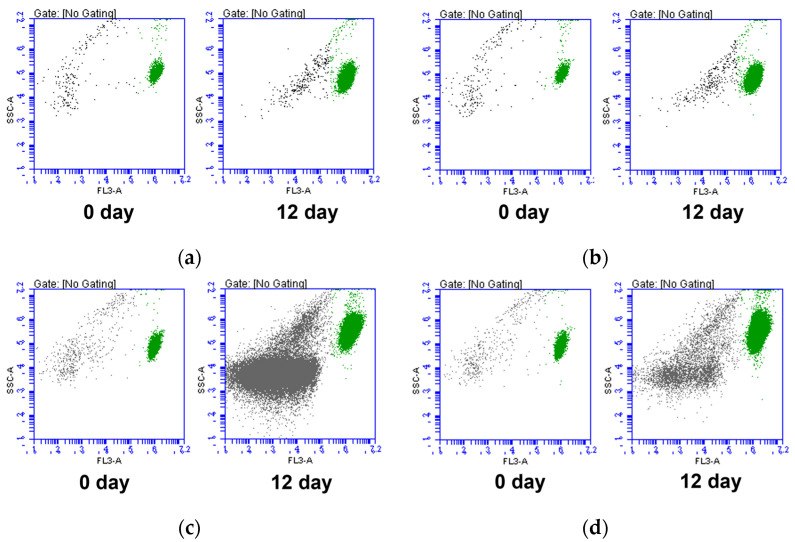
Cytograms of FL3-A vs. SSC-A of the *Tetraselmis* sp. culture medium during the cultivation periods based on flow cytometric measurement analyses. (**a**) Laboratory-scale of the conventional bubble column and (**b**) the ROSEMAX bioreactor. (**c**) Pilot-scale of the conventional bubble column and (**d**) the ROSEMAX bioreactor. Green and grey dots represent the *Tetraselmis* sp. live cells and cell debris, respectively.

During the cultivation period, we analyzed the microalgae cell size, auto-fluorescence, and cell complexity at both the laboratory and pilot scales (Figure 1). We confirmed that *Tetraselmis* sp. live cell clusters could be detected via the FL3-A channel. The clusters that were detected in the culture medium on the 0 day cytogram and that deviated from the FL3-A values could be evaluated as cell debris. Interestingly, at the pilot scale, we observed the emergence of clusters beyond *Tetraselmis* sp. live cells, in contrast to the laboratory scale. These clusters, including unknown non-photosynthetic microorganisms, extracellular substances, and cell debris, began to increase significantly in the conventional bioreactors compared to in ROSEMAX.

**Figure 2:**
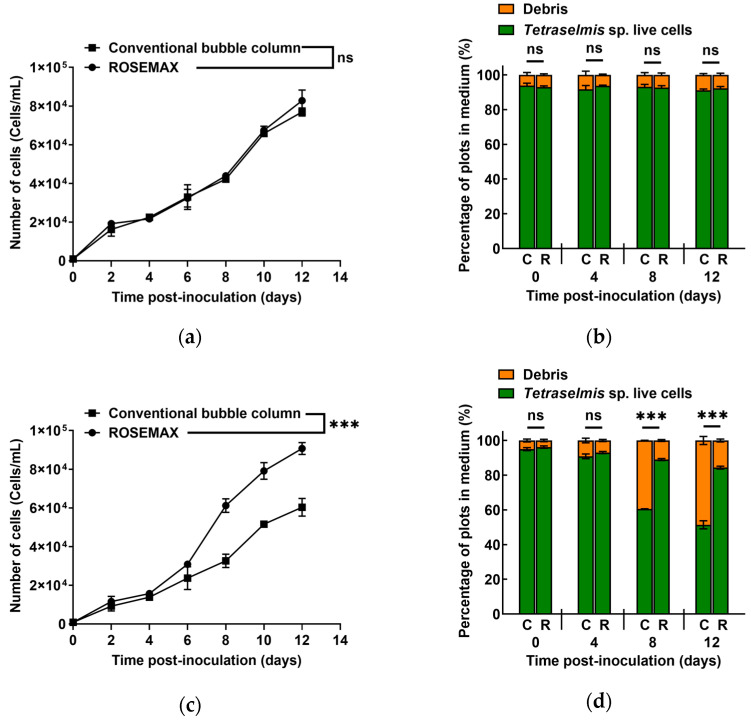
Number of *Tetraselmis* sp. cells cultured in the (**a**) laboratory and (**b**) pilot scale cultivations. The live cells proportion of *Tetraselmis* sp. in the culture medium in the (**c**) laboratory and (**d**) pilot scale cultivations during the culture periods. “ns” indicates no significant difference (*p* > 0.05). *** indicates a significant difference (*p* ≤ 0.05). C, conventional bubble column; R, ROSEMAX bioreactor.

Figure 2 illustrates that there was no significant difference in *Tetraselmis* sp. live cell counts between the two bioreactors during laboratory-scale operations (*p* > 0.05). However, when we scaled up to the pilot level, we observed a significant difference in *Tetraselmis* sp. live cell counts starting from the eighth day of cultivation (*p* ≤ 0.05).

At the laboratory scale, the proportion of *Tetraselmis* sp. live cells in a conventional bubble column and ROSEMAX showed values of 93.83 ± 1.41% and 93.06 ± 0.62% at the beginning of cultivation, respectively, and there was no significant difference on the final day (*p* > 0.05), with values of 91.17% ± 0.76 and 92.35 ± 0.99%. In contrast, at the pilot scale, both bioreactors showed a decrease in the proportion of live cells compared to that on the first day of cultivation. However, a significant difference between the two bioreactors was observed from the eighth day (*p* ≤ 0.05). Finally, the proportions of the live cells in a conventional bubble column and ROSEMAX were 51.46 ± 2.32% and 84.36 ± 0.84%, respectively, on the last day of cultivation. 

### 2.3. Impact on the Photosynthetic Performance

**Figure 3:**
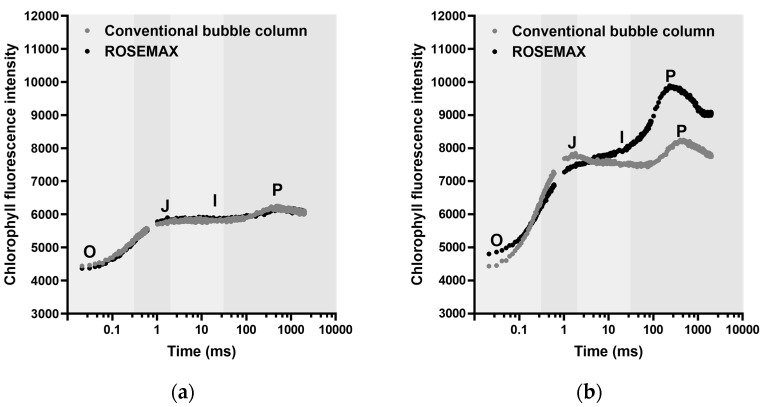
Changes in the chlorophyll fluorescence transient induction curve (OJIP curve) of *Tetraselmis* sp. cultivated at the pilot scale on the (**a**) first and (**b**) final day. Chlorophyll fluorescence intensities at 50 µs (O), 2 ms (J), 30 ms (I), and 500 ms to 1 s (P). Data are shown as the average of triplicates.

The OJIP curve indicates the performance of photosystem II within the cell. Chlorophyll fluorescence (OJIP) was used to investigate the photosynthetic activity of *Tetraselmis* sp. cultured in the conventional bubble column and ROSEMAX. Chlorophyll fluorescence transients were analyzed for a short time, covering O (50 µs), J (2 ms), I (30 ms), and P (500 ms to 1 s). As shown in Figure 3, the *Tetraselmis* sp. cells cultivated in the conventional bubble column and ROSEMAX bioreactors exhibited different OJIP curves, indicating variations in the photosynthetic performance.

**Figure 4:**
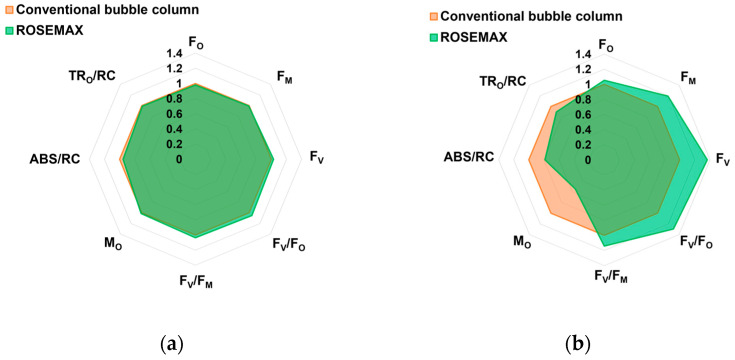
Spider plots showing the JIP-test parameters from chlorophyll-*a* fluorescence OJIP transient curves on the (**a**) first and (**b**) final day for *Tetraselmis* sp. cultivated at the pilot scale. Data are shown as the average of triplicates. Conventional bubble column, orange; ROSEMAX, green.

Figure 4 shows the specific physiological parameters based on previous OJIP fluorescence analyses. *Tetraselmis* sp. cultured in ROSEMAX showed increases in F_M_, F_V_, F_V_/F_O_, and F_V_/F_M_ by 1.19-, 1.36-, 1.29, and 1.14 times, respectively, compared to the conventional bubble column. However, the M_O_, ABS/RC, and TR_O_/RC decreased by 0.54, 0.79, and 0.90, respectively. Because there were no changes in F_O_, an increase in F_M_ resulted in an increase in F_V_.

## 3. Discussion

Variations in productivity and biochemical composition between laboratory- and pilot-scale outdoor-cultivated microalgal biomasses pose challenges to scaling up [38,39]. In this study, we aimed to evaluate a newly developed ROSEMAX bioreactor for the practical cultivation of *Tetraselmis* sp.

As shown in Table 1, there was no significant difference in the growth of *Tetraselmis* sp. cultured in the conventional bubble column and ROSEMAX at the laboratory scale (*p* > 0.05). However, at the pilot scale, the cell growth, and chlorophyll and carotenoid contents of *Tetraselmis* sp. cultured in ROSEMAX were significantly higher than those in the conventional bubble column (*p* ≤ 0.05). Similar to our results, previous studies on *Tetraselmis* sp. cultivation also showed a higher content of photosynthetic pigment in PBRs with enhanced biomass production [41]. However, excessive light conditions of more than 1000 µmol photons/m^2^/s can cause photosynthetic inhibition in *Tetraselmis* sp., leading to negative cell growth and decreased chlorophylls and carotenoids [42]. In this study, the pilot-scale culture dependent on natural light was performed within a light intensity of 67.24–457.50 µmol photons/m^2^/s, and photosynthetic inhibition was not a concern. The chlorophyll-*a* (14.60 ± 0.76 mg/g, DW) and total carotene (5.64 ± 0.81 mg/g, DW) contents of *Tetraselmis* sp. produced in this study were comparable to those of the representative microalgae *Spirulina platensis* and *Chlorella vulgaris*, containing 10.6 and 6.11 mg/g, DW of chlorophyll-*a* and 2.4 and 4.7 mg/g, DW total carotene content, respectively [7,43]. Photosynthetic efficiency (F_V_/F_M_) is positively correlated with photosynthetic pigments [44]. Therefore, in practice, there is a tendency to reduce the thickness of bioreactors to improve the photosynthetic efficiency of microalgae [45]. The chlorophyll fluorescence observed in the dark-adapted *Tetraselmis* sp. cells under a strong light pulse displayed an OJIP curve. Figure 3 shows the OJIP curves obtained from the minimum (F_O_) to maximum fluorescence (F_M_) [46]. The parameter F_V_/F_M_ serves as a representative indicator of photosynthetic performance, specifically reflecting the ability of photosystem II to absorb light energy under dark-adapted conditions, which is essential for the photosynthetic process [47,48]. Light availability refers to the amount of light accessible to photosynthetic organisms in an environment, and F_V_/F_M_ indicates the efficiency of light utilization in that environment. This study supports the idea that by increasing light accessibility in the central region of the photobioreactor, both biomass production and photosynthetic pigment content are likely to increase, as shown in our results (Figure 1 and Figure 4). 

Flow cytometry can separate cells with and without pigments (cell debris) using the autofluorescence of the photosynthetic pigments [32,49]. As shown in Figure 1, *Tetraselmis* sp. live cells were identified with high FL3-A signals and were distinguished from cell debris. Laboratory-scale cultivation conducted in an autoclaved medium with efficient fluid flow and an appropriate artificial light supply allowed both bioreactors to maintain a high proportion of live cells throughout the cultivation period. However, as shown in the cultivation results (Figure 1), increasing the culture scale led to an increase in the proportion of cell debris in the conventional bubble columns. Although this phenomenon is common, ROSEMAX exhibited relatively low levels of cell debris during the culture period. Figure 2 shows the results of the flow cytometric analysis in the laboratory- and pilot-scale cultivations. In the pilot-scale cultivation, the ratio of live cells cultured in the conventional bubble column bioreactor decreased as the cultivation progressed from 95.06 ± 0.85% to 51.46 ± 2.32% on the final day. In contrast, ROSEMAX maintained a live cell proportion of over 84.36 ± 0.84% until the end of the cultivation. 

**Figure 5:**
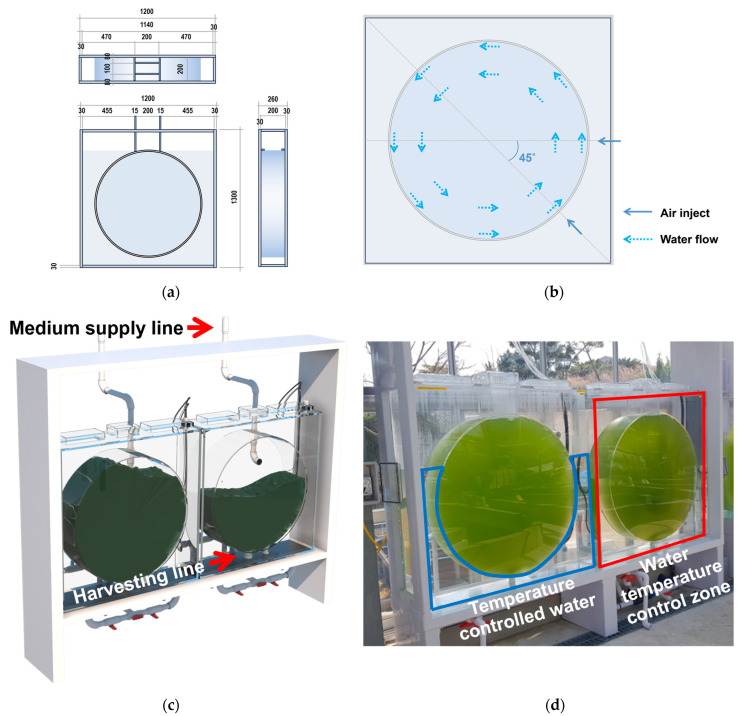
(**a**) Front, side, and top views, (**b**) schematic fluid flow, (**c**) 3D design, and (**d**) photograph of the ROSEMAX bioreactor used for the pilot-scale of *Tetraselmis* sp.

ROSEMAX was designed to overcome the disadvantages of the bubble column and airlift bioreactor. Airlift mixing involves the creation of a fluid flow by artificially separating the upward and downward flows in bubble columns [50]. To overcome the non-circulating zone of bubble columns during scale-up, transparent structures, such as baffles, are often added to bubble columns to physically enhance fluid flow [51,52]. However, installing baffles within a bioreactor can be costly and pose contamination issues, thus limiting its application in large-scale cultivation [53,54]. The structure of ROSEMAX allows for continuous fluid flow without a non-circulation zone and the need for attachments such as baffles. In the O-shaped ROSEMAX, injecting air at angles of 90° and 135° on one side creates an upward flow, whereas on the opposite side, between 180° and 360°, a downward flow naturally occurs along the curved shape of the reactor (Figure 5). Previous studies also improved the circulation in bioreactors by modifying the reactor structure without baffles [55]. To our knowledge, no bioreactor with a horizontal rotating cylindrical shape similar to ROSEMAX has been used for continuous microalgae cultivation. However, a similar tank shape, known as a Kreisel tank, is commonly used in jellyfish cultivation. The Kreisel tank creates a continuous rotating water flow to prevent plankton sedimentation [56], supporting the structural advantages of ROSEMAX. Therefore, a high proportion of *Tetraselmis* sp. live cells was achieved in ROSEMAX because of structural air mixing, which minimized the cell debris.

Photobioreactors are suggested to be designed with a thickness of 0.2 m or less, such as tubular and flat-panel photobioreactors, as this has been advantageously evaluated for large-scale production [57,58]. The pilot scale of ROSEMAX used in this study was in the form of a horizontally aligned cylindrical shape with a diameter of 1 m and a height of 0.2 m. This thickness is similar to that of general flat-panel and tubular reactors (0.2 m). Remarkably, despite ROSEMAX being scaled up approximately 36 times from its laboratory scale, there were no significant differences observed in biomass productivity and pigment content in the comparison between laboratory-scale and pilot-scale cultivations (Table 1) (*p* > 0.05). In contrast, the conventional bubble column bioreactor with a larger thickness (0.6 m) exhibited a substantial decrease in biomass concentration and photosynthetic pigment content. Additionally, the photosynthetic efficiency (F_V_/F_M_) in ROSEMAX increased relative to that in the conventional bubble column bioreactor as cultivation progressed. These results indicate that the efficient design of ROSEMAX allows microalgae to effectively utilize light, even during scaled-up cultivation. Furthermore, thin photobioreactors such as ROSEMAX can increase the surface area-to-volume ratio through vertical alignment [59,60,61]. However, when several such bioreactors are installed, it is generally recommended to limit the bioreactor height to 4 m or less to avoid affecting the light availability near the photobioreactors [57]. Taking this into consideration, if ROSEMAX were scaled up from a 1 m diameter to a 4 m diameter, it would allow microalgae cultivation at an industrial scale of approximately 2500 L. In addition, if the width were increased by the depth (40 cm) recommended for ORP [21], the volume of the ROSEMAX bioreactor would be approximately 5000 L. Although it is not included in this study, a mixing efficiency or mass transfer analysis related to cell mixing, which can make an unexpected problem at a larger scale, is required to scale-up to the scale mentioned above [62].

The results of this study demonstrate that ROSEMAX can be applied to industrial scales. During scaled-up cultivation from the laboratory scale to the pilot scale, no significant decrease in biomass concentration or photosynthetic pigment content was observed (*p* > 0.05). Following the pilot-scale cultivation, a noticeable difference in cell debris was observed between the biomass pellets cultivated in the conventional bubble column and ROSEMAX bioreactors. Therefore, we emphasize that the ROSEMAX bioreactor is suitable for reducing non-circulating zones in the culture medium and providing light accessibility, even in the central region of the bioreactor.

## 4. Materials and Methods

### 4.1. Strain and Culture Medium

The marine green microalgae *Tetraselmis* sp. KMMCC 1293 (=LIMS-PS-1293) was obtained from the Library of Marine Samples (LIMS, Geoje, Republic of Korea) at the Korea Institute of Ocean Science and Technology (KIOST, Busan, Republic of Korea). The culture medium used was modified Guillard’s f/2 medium [63] without vitamins [21] based on the underground seawater of Jeju Island. 

Seawater was disinfected with 1% (per culture volume) of a 13% sodium hypochlorite solution and neutralized with 1 mol/L sodium thiosulfate. Following this, 10% of the total volume of *Tetraselmis* sp. cells at a 0.2–0.3 g/L (DW) biomass concentration was inoculated into the flask or bioreactor.

### 4.2. Culture Conditions

Laboratory-scale culture conditions were maintained at 28.0 ± 1.0 °C under a 12 h:12 h light/dark cycle at 100 ± 5 µmol photons/m^2^/s of photosynthetic photon flux density (PPFD) with five white light-emitting diodes (LEDs) bars (DC 12 V, 7.2 Wm^−1^, LUMENLUX Co. Ltd., Bucheon, Republic of Korea). 

The pilot-scale cultivation was conducted in a glass greenhouse facility at the Jeju Bio Research Center of Korea Institute of Ocean Science and Technology (KIOST, Jeju Island, Republic of Korea). The air temperature inside the glass greenhouse ranged from 25.7 °C to 34.3 °C throughout the cultivation period. The water temperatures in the bioreactors varied from 25.94 °C to 28.18 °C without water control. Luminous was measured daily at 10 a.m. and 3 p.m. using a portable light meter (Li-250A, Li-COR, Lincoln, NE, USA), and showed daily variations within the range of 67.24–457.50 µmol photons/m^2^/s. Air was continuously injected at a 0.5 vvm aeration rate during the culture period.

### 4.3. Bioreactors

For the laboratory-scale cultivation, 5 L of Pyrex glass was used in the conventional bubble column. The pilot-scale bubble column, with a capacity of approximately 200 L (diameter 0.45 m, height 1.2 m), was constructed using acrylic material and contained 160 L of culture medium. 

The continuously circulated ROSEMAX bioreactor (Figure 5) consisted of two main water tanks: cultivation and external tanks. The cultivation tank was constructed from a transparent acrylic material and featured a cylindrical design. The laboratory scale culture ROSEMAX tank was 0.08 m in thickness and 0.3 m in diameter. The pilot-scale ROSEMAX was 0.2 m in thickness and 1 m in diameter. The cultivation tanks contained 5 L and 160 L of microalgal cells. To circulate the culture medium within the cultivation tank, air was injected through six air-injection ports, with three ports positioned at angles of 90° and the other three ports positioned at 135°. The culture medium within the tank was circulated counterclockwise. As shown in Figure 5a, the top of the cultivation tank has a rectangular passage that leads out of the bioreactor, which is necessary for microalgal cell inoculation and sampling. The bottom of the cultivation tank was equipped with a drain system at its center to harvest the culture medium. Additionally, a fresh culture medium supply line was installed at the rear of the bioreactor to enable sustainable culture medium replenishment. 

The external tank (water temperature control zone in Figure 5d), made of acrylic in a rectangular shape, featured a temperature-controlled circulation pump to regulate the temperature of the external tank water. This design allows for temperature control within the isolated space and extends the control of the internal cultivation tank temperature.

### 4.4. Biomass Concentration 

The dried *Tetraselmis* sp. biomass concentration was calculated based on the difference in dry weight before and after filtration. A 20 mL sample of the culture medium was filtered using a 47 mm diameter GF/C filter paper (Whatman, Maidstone, UK). The filter paper was subsequently dried at 50 °C in a drying oven for a duration of 12 h to obtain the dried cell weight (g/L). 

### 4.5. Cell Harvesting and Phytochemical Analysis

Following the laboratory-scale cultivation, *Tetraselmis* sp. were harvested using centrifugation at 9000× *g* for 20 min at 4 °C (Sorvall RC6 Plus; Thermo Fisher Scientific, Asheville, NC, USA). The cultured pilot-scale cells were harvested using a continuous-flow industrial tubular centrifuge (GQLY series, Hanil S.M.E, Anyang, Republic of Korea). The wet biomass was then frozen at −70 °C for 24 h and lyophilized in a freeze dryer (FDTA-45; Operon, Gimpo, Republic of Korea) for 48 h. The dried biomass was packed in a shaded bag and stored at −70 °C until further analysis. 

To analyze the contents of chlorophyll-*a*, chlorophyll-*b*, protochlorophyllide, and total carotene, 50 mg of dried powder of the *Tetraselmis* sp. was mixed with 5 mL of 90% acetone. The mixture was sonicated for 10 min and then stored at 4 °C for 30 min. It was then centrifuged for 20 min at 3000 rpm at 4 °C (Sorvall RC6 plus, Thermo Fisher, Asheville, NC, USA) and the supernatant was collected. The extraction was repeated until no more photosynthetic pigments were extracted. The absorbance of the extracts was determined using an ultraviolet–visible spectrophotometer (Optizen POP Bio, Mecasy Co. Ltd., Daejeon, Republic of Korea) at 470, 625, 647, and 664 nm.

Optical densities (ODs) were calculated for the chlorophyll-*a*, chlorophyll-*b*, protochlorophyllide, and total carotene contents using the following equations [64]:

Chlorophyll-*a* = 12.65 × OD 664 − 2.99 × OD 647 − 0.04 × OD 625 (1)

Chlorophyll-*b* = −5.48 × OD 664 + 23.44 × OD 647 − 0.97 × OD 625 (2)

Protochlorophyllide = −3.49 × OD 664 − 5.25 × OD 647 + 28.3 × OD 625(3)

Total carotene = (1000 × OD 470 − 1.280 Chl*a* − 56.7 Chl*b*)/230 (4)

### 4.6. Photosynthetic Performance

The photosynthetic performance of *Tetraselmis* sp. cultivated in the conventional bubble column bioreactor and ROSEMAX was monitored every 2 days using a pulse-amplitude magnitude fluorometer (AquaPen-C, AP110/C; Photon Systems Instruments, Drasov, Czech Republic). Before the measurement, 20 mL of microalgae samples were collected and adapted in the dark for 20 min at room temperature. After adjusting the samples to an absorbance of 0.3 (750 nm), 3 mL of dark-adapted samples were added to a 4 mL cuvette with a 10 mm light path for measurement.

This measurement included the analysis of the chlorophyll-*a* fluorescence induction (OJIP) curves, initial fluorescence intensity (F_O_) and maximum fluorescence intensity (F_M_) of the dark-adapted cells, maximal variable fluorescence (F_V_ = F_M_ − F_O_), maximum quantum yield (F_V_/F_M_), net closing rate of the reaction center (M_O_), trapping flux (TR_O_/RC), and effective antenna size expressed as the absorbance per reaction center (ABS/RC) during a 10 s saturating light pulse of 3000 µmol photons/m^2^/s at a 620 nm emission. AquaPen-C recorded the fluorescence intensities at 50 µs (O or F_O_), 2 ms (J), 30 ms (I), and 500 ms to 1 s (P or F_M_) as the OJIP test kinetics.

### 4.7. Flow Cytometric Measurement

The flow cytometry analysis of the *Tetraselmis* sp. cells was performed using a flow cytometer (BD Accuri C6 Accuri Cytometers, Inc., Ann Arbor, MI, USA). We analyzed the *Tetraselmis* sp. live cell population and the extent of cell mortality due to sedimentation in the microalgae biomass using flow cytometry. We collected 50 μL of culture medium and 10 μL was injected for analysis with a core size of 22 μm at a 66 µL/min flow rate. During this process, we detected the signals at the FSC (cell size), SSC-A (cell granularity), and FL3-A channel (>670 ± 25 nm; chlorophyll autofluorescence). Prior to the study, pure cultured *Tetraselmis* sp. cells were analyzed and gated. The cells that had lost pigments or became cell debris were detected outside the gate.

### 4.8. Statistical Analysis

All the data were presented as the mean ± standard deviation. All the statistical analyses were performed using the GraphPad Prism 8.4.3 software (GraphPad, San Diego, CA, USA). The values of each treatment were analyzed using a Student’s two-tailed *t*-test for unpaired data. Statistical significance was set at *p* values ≤ 0.05.

## 5. Conclusions

This study shows the potential of the ROSEMAX bioreactor as an ideal bioreactor for cultivation of *Tetraselmis* sp. biomass and photosynthetic pigment production. The cultivation of *Tetraselmis* sp. is essential to meet the consumer demands for bioactive compounds, and the pilot-scale ROSEMAX offers reliable product quality and quantity compared to conventional bubble columns. In pilot scale cultivations, the biomass concentration, chlorophyll-*a* content, and total carotene content of *Tetraselmis* sp. cultivated in ROSEMAX were significantly (1.44 times, 1.39 times, and 1.74 times, respectively) higher than those in conventional bubble columns (*p* ≤ 0.05). A flow cytometry analysis showed that the live cell proportion in ROSEMAX was 84.36 ± 0.84% on the final day of cultivation, while that in conventional bubble columns was 51.46 ± 2.32%. Photosynthetic efficiency was 1.14 times higher in ROSEMAX. These results highlight ROSEMAX’s ability to prevent cell sedimentation and non-circulating zones during large-scale microalgae production. Notably, ROSEMAX’s thin and efficient design allows for effective light utilization, ensuring a consistent biomass concentration and photosynthetic pigment content, even in scaled-up cultivations. 

## Figures and Tables

**Table 1:** Comparison of the biomass concentration and photosynthetic pigment content of *Tetraselmis* sp. cultured in both laboratory and pilot scale cultivations. Capital letters denote significant differences between the conventional bubble column bioreactor and ROSEMAX, while lowercase letters represent significant distinctions between the laboratory scale and pilot scale, determined with a Student’s *t*-test (*p* ≤ 0.05).

	Conventional Bubble Column	ROSEMAX
Laboratory scale (5 L)		
Biomass concentration (g/L, DW)	1.71 ± 0.09 ^a^	1.89 ± 0.14
Chlorophyll-*a* (mg/g, DW)	14.75 ± 1.25 ^a^	15.79 ± 1.17
Chlorophyll-*b* (mg/g, DW)	7.58 ± 0.22 ^Ba^	8.84 ± 0.47 ^Aa^
Protochlorophyllide (mg/g, DW)	3.02 ± 0.27 ^a^	2.82 ± 0.40
Total carotene (mg/g, DW)	6.08 ± 0.65 ^a^	6.07 ± 0.21
Pilot scale (160 L)		
Biomass concentration (g/L, DW)	1.17 ± 0.11 ^Bb^	1.69 ± 0.11 ^A^
Chlorophyll-*a* (mg/g, DW)	10.67 ± 0.72 ^Bb^	14.60 ± 0.76 ^A^
Chlorophyll-*b* (mg/g, DW)	5.87 ± 0.54 ^Bb^	7.64 ± 0.09 ^Ab^
Protochlorophyllide (mg/g, DW)	0.84 ± 0.49 ^Bb^	2.28 ± 0.41 ^A^
Total carotene (mg/g, DW)	3.24 ± 0.56 ^Bb^	5.64 ± 0.81 ^A^

## Data Availability

The data presented in this study are available on request from the corresponding author.
